# Lossless Compression of Large Field-of-View Infrared Video Based on Transform Domain Hybrid Prediction

**DOI:** 10.3390/s26030868

**Published:** 2026-01-28

**Authors:** Ya Liu, Rui Zhang, Yong Zhang, Yuwei Chen

**Affiliations:** 1 Shanghai Institute of Technical Physics, Chinese Academy of Sciences, Shanghai 200083, China; liuya182@mails.ucas.ac.cn; 2 University of Chinese Academy of Sciences, Beijing 100049, China; 3 Hangzhou Institute for Advanced Study, University of Chinese Academy of Sciences, Hangzhou 310024, China; yuwei.chen@nls.fi

**Keywords:** large field-of-view infrared video, lossless compression, discrete wavelet transform, multi-view high efficiency video coding (MV-HEVC), optimal direction prediction

## Abstract

Large field-of-view (FOV) infrared imaging, widely utilized in applications including target detection and remote sensing, generates massive datasets that pose significant challenges for transmission and storage. To address this issue, we propose an efficient lossless compression method for large FOV infrared video. Our approach employs a hybrid prediction strategy within the transform domain. The video frames are first decomposed into low- and high-frequency components via the discrete wavelet transform. For the low-frequency subbands, an improved low-latency Multi-view High-Efficiency Video Coding (MV-HEVC) encoder is adopted, where the background reference frames are treated as one view to enable more accurate inter-frame prediction. For high-frequency components, pixel-wise clustered edge prediction is applied. Furthermore, the prediction residuals are reduced by optimal direction prediction, according to the principle of minimizing residual energy. Experimental results demonstrate that our method significantly outperforms mainstream video compression techniques. While maintaining compression performance comparable to MV-HEVC, the proposed method exhibits a 19.3-fold improvement in computational efficiency.

## 1. Introduction

Infrared imaging technology, with its unique imaging mechanism, has found extensive applications in in various fields, including target recognition [1,2], remote sensing observation [3,4,5], security monitoring [6], and industrial inspection [7]. The large FOV scanning imaging system enables coverage of a wide field through a push-scan mechanism. In satellite remote sensing, this technology reduces data acquisition cycles and significantly improves the efficiency of acquiring surface information over large areas. Similarly, in infrared search systems, the detection system captures 360° panoramic infrared images in the horizontal direction via push-scanning, facilitating wide-field omnidirectional monitoring. Nevertheless, the rapid growth in the volume of image data generated by such high-resolution, wide-field imaging systems has created serious challenges for the storage and transmission of infrared data. Thus, efficient coding methods for compressing large FOV infrared videos are required to address these challenges.

Existing compression techniques can be broadly categorized into lossy compression [8,9] and lossless compression [10,11,12]. Lossless compression allows for the perfect reconstruction of the original data, whereas lossy compression results in a certain degree of information loss. This paper focuses on lossless compression techniques, as ensuring data integrity is essential for many wide-field infrared video applications. Unlike visible-light images, most modern infrared sensors capture high-bit-depth images, with each pixel typically represented by 14–16 bits. Traditional standards and algorithms for lossless compression of high-bit-depth infrared video include Motion JPEG2000 [13], H.264/AVC [14], H.265/HEVC [15], H.266/VVC [16], JPEG2000 [17], JPEG-XT [18], JPEG-LS [19,20], PNG, and Gzip [21], among others. Early video compression methods, such as Motion JPEG2000, perform independent compression on each frame of the video. This approach does not account for temporal correlations between frames, resulting in relatively low compression ratios. Later advancements, such as the H.26x series, employ intra- and inter-prediction techniques to more effectively reduce spatial and temporal redundancies. Notably, the H.266/VVC standard achieves remarkable bit rate reductions of approximately 50% over H.265/HEVC and 75% over H.264/AVC. However, this efficiency comes at the cost of a significant increase in encoding complexity [22,23], presenting significant challenges for practical deployment in large-format infrared images.

As a representative transform-based compression method [24,25], JPEG 2000 exhibits significant advantages for image compression tasks. It employs DWT with either a 5/3 or 9/7 wavelet filter as its core transformation technique, decomposing the image into subbands of varying orientations and resolutions. Compared to JPEG [26,27], JPEG2000 achieves a significantly higher compression ratio. Building on JPEG, JPEG-XT is backward compatible with the JPEG compression standard and has been applied in numerous fields. It utilizes a two-layer bit-depth scalable coding approach [28,29], wherein the original high-bit-depth infrared image is split into two layers after tone mapping. The first layer consists of an 8-bit low dynamic range (LDR) image, compatible with standard 8-bit displays, while the second layer contains residuals between the original image and the inverse tone-mapped LDR image. However, to achieve superior lossless compression performance, it requires determining encoding parameter values for each image to be encoded, which increases computational complexity. On the other hand, JPEG-LS supports both lossless and near-lossless compression with relatively low complexity, enabling faster encoding speeds. It compresses images either through a regular prediction mode with context modeling, pixel prediction, and prediction error encoding, or through run-length encoding for smooth regions. However, this approach mainly focuses on horizontal and vertical diagonal edges; its relatively simple prediction model is insufficient for accurately capturing complex regions in infrared images.

In recent years, deep learning-based video compression methods [30,31,32] have shown significant potential by leveraging the powerful learning capabilities of neural networks. The scope of these learning paradigms has expanded rapidly, with recent work demonstrating that quantum convolutional neural networks [33] can enhance feature extraction in image generation tasks. Drawing on such advanced representation capabilities, deep learning-based compression methods leverage end-to-end [34] optimization to capture spatial and temporal redundancies effectively, surpassing many limitations of traditional techniques. However, their practicality remains constrained by challenges such as high computational costs and deployment difficulties.

Large FOV infrared videos exhibit the following key characteristics: (1) Unlike visible light images rich in texture and color, infrared images are characterized by lower contrast, a lack of chrominance components, and specific noise patterns dominated by thermal sensors [35,36]. Traditional compression standards, such as JPEG2000 and H.264, are primarily oriented towards RGB images and do not account for the specifics of infrared data [37]; (2) the majority of the background remains static, with only a small portion containing moving regions; (3) the resolution is extremely high, with periodic scanning of the same scene generating significant repetitive background information, resulting in massive data volumes. Nevertheless, existing methods lack the ability to balance the data volume and computational demands inherent in large FOV infrared video compression. Traditional intra-frame image compression methods are ineffective at reducing inter-frame redundancy in videos, leading to poor compression ratios. Meanwhile, video coding standards based on inter-frame prediction, such as the H.26x series, limit the number of reference frames, restricting their ability to exploit similarities between the current frame and frames from previous scanning cycles of the same scene. These standards only reduce redundancy within short-term, adjacent temporal sequences, failing to effectively leverage background information in large FOV videos, and are therefore unsuitable for ultra-high-resolution infrared videos. Therefore, there remains a lack of coding schemes capable of exploiting the long-term background redundancy in large FOV videos while avoiding the excessive computational costs associated with standard video codecs.

We focus on effectively exploiting background redundancy in large FOV videos by integrating transform-domain processing with an efficient predictive coding strategy. Specifically, high-resolution infrared images are first divided into sub-images and then transformed into the frequency domain via DWT. A targeted prediction method tailored to the characteristics of the frequency domain is proposed. Specifically, the set of low-frequency components in the background reference frame queue is designated as the background reference view, while the set of target images is defined as the view to be encoded. For the low-frequency components, we employ an improved dual-view H.265/HEVC encoder with a forward prediction structure, which fully leverages the inter-frame redundancy of background reference frames. For high-frequency components, we propose a pixel-wise clustering-based edge prediction method integrated with the low-complexity JPEG-LS algorithm. Furthermore, an optimal directional prediction strategy is adopted to further reduce the prediction residual energy, thereby enhancing compression efficiency. The main contributions of the paper are the following:

(1) We propose a systematic hybrid coding framework tailored for large FOV infrared videos. By decomposing images into frequency subbands via integer lifting wavelet transform, we apply differentiated prediction strategies specifically to low-frequency backgrounds and high-frequency edges, striking an optimal balance between compression efficiency and computational complexity.
(2) We introduce an innovative dual-view prediction mechanism for low-frequency components. By constructing a background reference frame queue and creatively adapting the MV-HEVC architecture for single-view infrared video, we treat the background reference and current frame as two views. This allows us to effectively exploit long-term inter-frame redundancy without the high computational cost of full motion estimation.
(3) We design a specialized pixel-wise clustering-based edge predictor for high-frequency components. By incorporating the Sorted Equipartition-based Single-Iteration Clustering (SESIC) algorithm, reference pixels are grouped by intensity similarity to enable inter-frame prediction without additional side information and the computational cost of motion estimation. Furthermore, an optimal directional prediction strategy is applied to further reduce residual energy in texture-complex regions.

The remainder of this paper is organized as follows: Section 2 reviews existing related work on background-redundant video compression and mainstream video compression standards. Section 3 presents the proposed low-frequency and high-frequency compression method for large FOV infrared video. Section 4 provides performance evaluation and complexity comparison for different image coding algorithms. Section 5 concludes this paper.

## 2. Related Works

### 2.1. Image Decomposition and Spatial Prediction Techniques

To analyze image signals more effectively, images are often decomposed into different frequency subbands. The theoretical basis for this decomposition lies in the distinct statistical characteristics of image components. Low frequencies typically represent smooth background structures and gradual changes, while high frequencies capture edges, fine details, and noise. As noted by Wiseman [38], separating these components enables processing that aligns with the frequency-dependent characteristics of human perceptual systems. Building on this theoretical foundation, differentiated coding strategies tailored to the distinct characteristics of each frequency band can be leveraged to achieve improved compression efficiency.

To implement this decomposition, the Discrete Wavelet Transform (DWT) has become widely preferred in modern compression frameworks, most notably the JPEG 2000 standard [17], owing to its superior energy compaction capability and its theoretical foundation in multiresolution analysis [39]. Furthermore, recent studies by Vujović et al. [40] indicate that the wavelet transform is effective in identifying localized features in complex signals and can accurately capture fine-grained features in data.

Spatial prediction techniques capitalize on intra-frame correlations by estimating the current pixel value from previously encoded neighboring pixels, thereby mitigating spatial redundancy without reliance on temporal frame information. Leading lossless compression standards, including JPEG-LS [19], adopt the Median Edge Detection (MED) predictor, which infers pixel intensity based on the local gradient properties of adjacent samples. Similarly, CALIC [41] introduces the Gradient-Adjusted Predictor (GAP), which constructs a more refined context model to enhance the fidelity of pixel prediction. A critical advantage of these gradient-based prediction frameworks is their inherently low computational complexity, which renders them well-suited for low-latency real-time compression tasks.

### 2.2. Related Research on Background Redundancy Removal in Video

Based on video image compression standards, numerous studies focus on addressing background redundancy in video coding. For instance, Refs. [42,43,44,45,46] construct background reference models for surveillance videos. Belyaev and Forchhammer [47] created aerial maps from reconstructed frames to serve as inter-frame references, thereby compressing UAV infrared video using an MV-HEVC [48] codec. The most relevant reference frames are selected from the historical video database and designated as historical reference background frames to eliminate long-term background redundancy in I-frames of UAV videos [49]. A background reference library based on Google Earth data was developed to effectively address long-term background redundancy in satellite videos [50,51]. Some methods exploit the global view redundancy in video datasets. For instance, Yue et al. [52] utilized SIFT features to search for similar blocks within reference image datasets for encoding. Zuo et al. [53] proposed a library-based coding scheme specifically designed for videos with repetitive scenes, thereby enhancing coding performance. Similarly, Ma et al. [54] built a vehicle and background library, enabling the coding of traffic surveillance videos by retrieving similar vehicles and backgrounds from the library.

All of these approaches rely on the strong correlations between similar images to reduce redundant background information. However, the unique characteristics of large FOV infrared videos, such as their ultra-wide field, ultra-high resolution, and distinctive redundancy patterns, introduce significant challenges for these methods. As a result, existing approaches struggle to adapt and are unsuitable for compressing such videos.

### 2.3. Reference Frames of the H.26X Series

Both the H.264 and H.265 video compression standards employ multi-frame reference techniques [55] for inter-frame prediction, which improve accuracy and achieve higher coding efficiency. These standards incorporate both short-term and long-term reference frames. Short-term reference frames are primarily used to predict adjacent frames over short time intervals, whereas long-term reference frames enable inter-frame prediction over extended time periods. Images stored in the reference frame queue serve as inter-frame references for subsequent encoding stages, reducing temporal redundancy between frames.

In video encoders, the computational complexity of inter-frame motion estimation accounts for the largest proportion of overall complexity [56,57]. The number of reference frames significantly impacts encoder performance: a higher number of reference frames provides more inter-frame reference information, which helps enhance encoding efficiency. However, this comes at the cost of increased encoder complexity. Thus, it is crucial to select an optimal number of reference frames during encoding to balance compression efficiency and the added complexity introduced by a large number of reference frames.

## 3. Methods

### 3.1. Video Characteristics and Encoding Approach

#### 3.1.1. Characteristics of Large FOV Infrared Video

**Figure 1:**
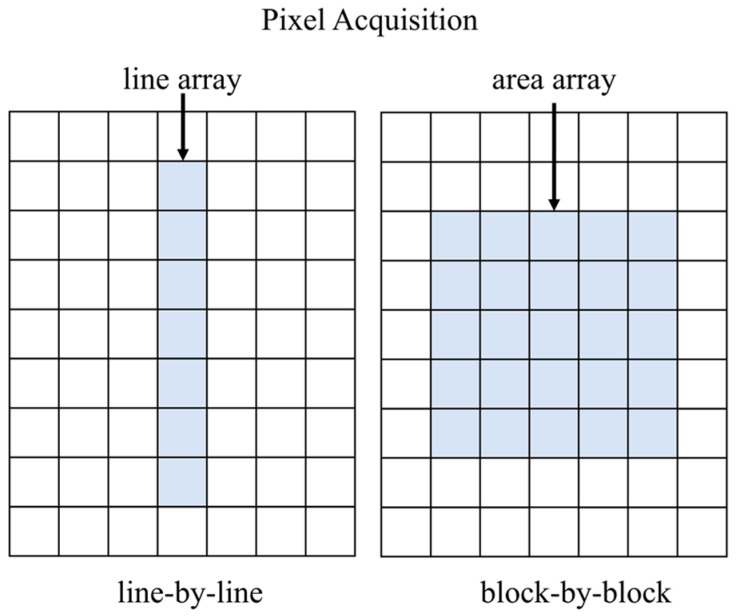
Methods of infrared image acquisition. Line array for line-by-line acquisition, area array block-by-block acquisition.

As shown in Figure 1, large FOV infrared images can be acquired through two primary methods: one involves a long linear array detector, and the other employs an area array detector. The detector performs periodic scanning by rotating on a turntable, enabling the acquisition of 360° horizontal panoramic infrared video with a wide field of view and high resolution.

**Figure 2:**
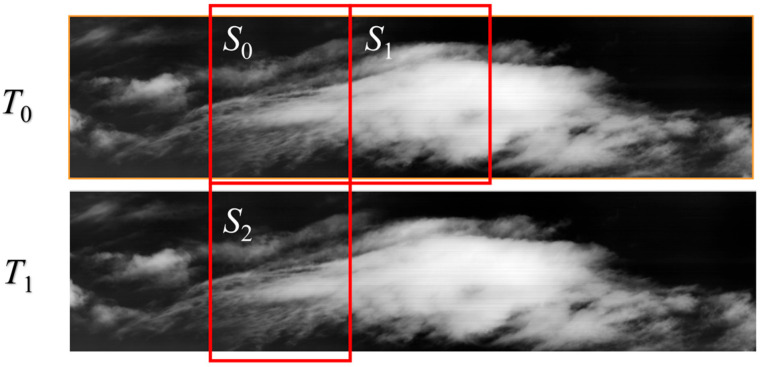
Partial of images in a large FOV infrared video.

Figure 2 presents a partial representation of images from two adjacent push-scan periods (*T*_0_ and *T*_1_), which illustrate the correlation characteristics of large FOV. Specifically, two sets of image blocks are highlighted: one set consists of blocks *S*_0_ and *S*_1_, which are temporally adjacent during acquisition; the other set includes blocks *S*_0_ and *S*_2_, which originate from different push-scan periods but correspond to the same spatial orientation.

A key observation from these blocks is their distinct correlation levels: the scenes in *S*_0_ and *S*_1_ differ significantly, resulting in weak temporal correlation. In contrast, *S*_0_ and *S*_2_ share similar scenes, exhibiting strong orientation-based correlation. This comparison reveals that periodic push-scanning systems generate substantial orientation-based background redundancy—an important characteristic that could be exploited for compression. However, traditional coding standards fail to exploit this redundancy effectively.

Both H.264 and H.265 are constrained by two critical limitations. First, the number of allowable reference frames is restricted, which limits the ability to capture orientation-based correlations and reduce background redundancy in wide-field scenarios. Second, even when reference frames are increased, the computational complexity of inter-frame motion estimation grows significantly. These limitations suggest that the traditional multi-reference frame techniques of H.264 and H.265 are ill-suited for compressing ultra-high-resolution, large FOV infrared videos.

Consequently, the critical challenge addressed in this study is to effectively exploit the strong orientation-based correlation among similar images to reduce background redundancy, while minimizing the associated encoding time.

#### 3.1.2. Encoding Approach

**Figure 3:**
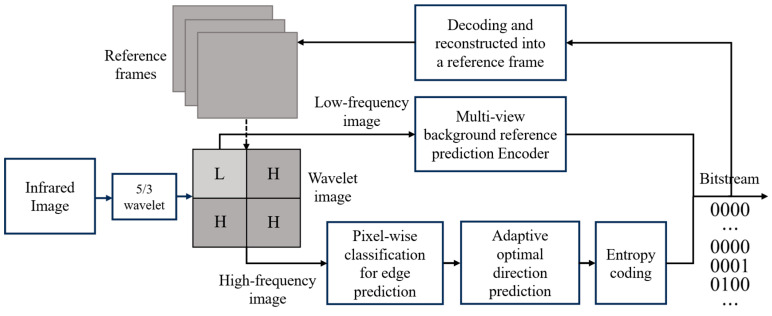
The proposed coding flowchart.

Let $I_{t}$ denote the current input frame of the large FOV infrared video at time instance t. The proposed framework (Figure 3) integrates three key modules into a synergistic hybrid structure to effectively balance compression performance. The complete implementation logic of the compression framework is summarized in Algorithm 1.

   **Algorithm 1**: Overall compression framework for large FOV infrared video   **Input**: Infrared video frames $\left\{I_{1},I_{2},\ldots ,I_{n}\right\}$               Q_capacity: Capacity of background reference queue (matching sub-images perpush-scan period)              Wavelet decomposition level: l=3   **Output**: Compressed video bitstream: B          1.      // Step 1: Initialize background reference queue          2.      $Q_{ref}$ ← Empty Queue (FIFO, capacity = Q_capacity)          3.      Bitstream ← Empty
          4.      // Step 2: Preprocess and encode each frame          5.      for each frame $I_{t}$ in video sequence do:          6.             // Step 2.1: 3-level 5/3 integer lifting wavelet decomposition          7.             $\left\{L_{t},\;H_{t}\}\leftarrow \Psi (I_{t},l\right)$ // Ψ(⋅) wavelet transform function, split into low/high frequency
          8.             // Step 2.2: Encode low-frequency subband          9.             **if**
$Q_{ref}$ is not empty **then**          10.                  $V_{1}$ ← Extract low-frequency components from $Q_{ref}$ (reference view)          11.                   $V_{2}$ ← $L_{t}$ (coding view, low-frequency of current frame)          12.                  Encode $V_{1}$ as I-frames          13.                  Encode $V_{2}$ as P-frames (forward prediction: ${\hat{L}}_{t}=P_{inter}(L_{t}|V_{1})$          14.                  Compute residual: $R_{L}=L_{t}-{\hat{L}}_{t}$          15.                  Append $R_{L}$ entropy coding bitstream $B_{L}$ to Bitstream          16.             **end if**
          17.             // Step 2.3: Encode high-frequency subband          18.             $H_{ref}$ ← Extract high-frequency component from $Q_{ref}$ (background reference)          19.             // Predict $H_{t}$ using pixel-wise clustering:          20.             ${\hat{H}}_{t}$← EdgeAdaptivePrediction($H_{t}$, $H_{ref}$)          21.             Compute residual: $R_{H}=H_{t}-{\hat{H}}_{t}$          22.             //Apply adaptive directional prediction to $R_{H}$:          23.             ${\hat{R}}_{H}$ = DirectionalResidualPrediction($R_{H}$)          24.             // Encode residuals using modified JPEG-LS:          25.             $B_{H}$ = m_JPEG-LS(${\hat{R}}_{H}$)          26.             Append entropy coding bitstream $B_{H}$ to Bitstream
          27.             // Step 2.4: Update background reference queue (FIFO)          28.             **if** Length($Q_{ref}$) ≥ Q_capacity **then**          29.                  Dequeue the oldest frame from $Q_{ref}$          30.             **end if**          31.             Enqueue $I_{t}$ into $Q_{ref}$          32.      **end for**
          33.      // Step 3: Output final compressed bitstream          34.      return Bitstream

1. Frequency Separation (5/3 Integer Lifting Wavelet Transform)

Before encoding, the large FOV infrared video is divided into sub-images. A 5/3 Integer Lifting Wavelet Transform, denoted as Ψ(⋅), is applied to decompose the frame $I_{t}$ into low-frequency $\left(L_{t}\right)$ and high-frequency $\left(H_{t}\right)$ subbands $\left\{L_{t},\;H_{t}\}=\Psi (\cdot \right)$, enabling tailored predictive encoding for each component.

2. Low-Frequency Processing (Adapted MV-HEVC)

Reconstructed images are stored in a background reference queue, denoted as $Q_{ref}=\{{\hat{I}}_{t-N},\;{\hat{I}}_{t-N+1},\cdots ,{\hat{I}}_{t-1}\}$, and their low-frequency subbands $L_{ref}$ serve as a reference view to generate the predicted low-frequency component ${\hat{L}}_{t}=P_{inter}(L_{t}|{\;L}_{ref})$, where $P_{inter}(\cdot )$ represents the inter-frame prediction function. This mechanism allows efficient exploitation of long-term background correlations while reducing motion estimation complexity due to lower resolution.

3. High-Frequency Processing (Pixel-Wise and Directional Prediction)

Pixel-wise edge prediction is applied to high-frequency components by utilizing the SESIC algorithm to dynamically cluster pixels within the high-frequency reference frame $H_{ref}$, combined with optimal directional prediction to further reduce residual energy: $\;{{\hat{H}}_{t}=D_{opt}(SESIC(H}_{t}\;|\;H_{ref}))$. This approach avoids side information and complex motion search, significantly lowering encoding latency.

### 3.2. Data Pre-Processing

#### 3.2.1. Constructing the Reference Frame Queue

**Figure 4:**
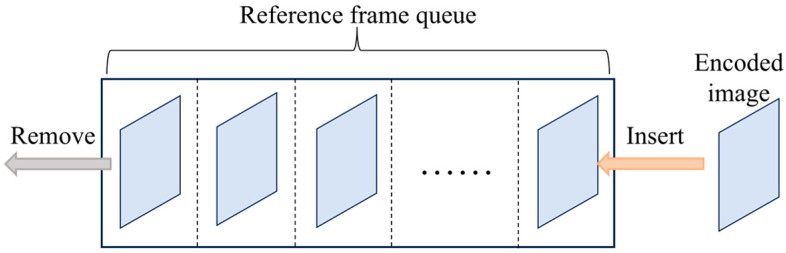
The reference frame queue.

As shown in Figure 4, this study constructs a reference frame queue with a capacity matching the number of sub-images in one push-scan period. The queue operates based on the first-in, first-out (FIFO) principle, storing images in their encoding order. During inter-frame prediction, encoded images from the queue are selected as inter-frame references. After encoding the current image, the oldest reference frame is removed from the queue, and the newly encoded image is appended to its end.

#### 3.2.2. Multi-Resolution Integer Lifting Wavelet Decomposition

The DWT decomposes a signal into approximation and detail coefficients at multiple scales, allowing for multi-scale signal analysis. When applied to images, the non-integer wavelet transform produces floating-point coefficients, and direct encoding of these coefficients may result in information loss. To ensure lossless compression, this study employs the 5/3 integer lifting wavelet transform [58] (Equations (1) and (2)), in which, $d_{n}$ and $c_{n}$ represent the high-frequency and low-frequency components of the image, respectively.

(1) $$d_{n}=X_{2n+1}-\lfloor (X_{2n}+X_{2n+2})/2\rfloor$$

(2) $$c_{n}=X_{2n}+\lfloor (d_{n-1}+d_{n}+2)/4\rfloor$$

The number of wavelet transform levels is a critical parameter balancing compression performance and computational complexity. To determine the optimal level, we conducted experiments on multi-frame images, analyzing the residual error between the current frame and the background reference frame after transformation. The optimal number of levels is determined by minimizing the Mean Absolute Difference (MAD), as shown in Equation (3). The MAD values for each level are compared, where a smaller MAD indicates smaller residual errors between image frames after transformation.

(3) $$MAD(l)=\frac{1}{M\times N}[\sum_{x=1}^{M}\sum_{y=1}^{N}W_{i}(x,y,l)-W_{i-1}(x,y,l)]$$

where M and N represent the image size; $W_{i}$ and $W_{i-1}$ denote the image to be encoded and the background reference frame after wavelet transform, respectively; and l denotes the number of wavelet transform levels.

We evaluated decomposition levels from l=0 to l=4. The experimental results, detailing the MAD and the reduction in error relative to the previous level, are presented in Table 1.

According to the experimental results, the MAD decreases significantly from Level 0 to Level 2. At Level 3, the MAD reaches 2.88, which is extremely close to the value at Level 4.

**Figure 5:**
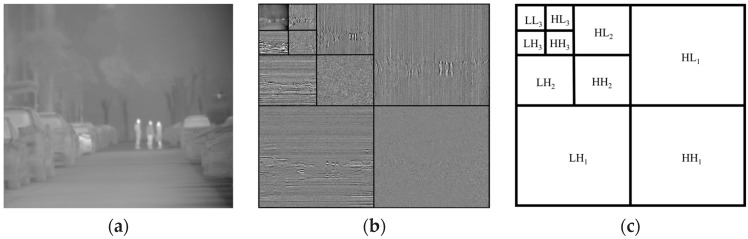
The original image and 3-level DWT. (**a**) The original image; (**b**) the 3-level DWT image; (**c**) the subband distribution after DWT.

The gain in increasing from Level 3 to Level 4 is negligible, whereas the computation for the lifting steps increases with each additional level. Consequently, to achieve a favorable trade-off between coding efficiency and computational complexity, we selected Level 3 as the standard decomposition depth for this study. The original image, the 3-level DWT image, and the subband distribution after DWT are shown in Figure 5a–c.

### 3.3. Low-Frequency Dual-View Prediction

**Figure 6:**
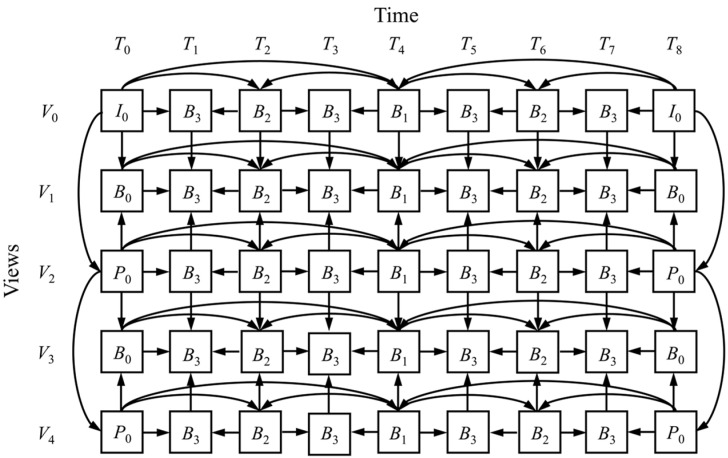
MVC reference prediction structure.

Multi-view video [59,60] refers to video captured simultaneously by multiple cameras positioned at different angles of the same scene. In addition to temporal and spatial redundancies, multi-view video also contains inter-viewpoint redundancies. The MV-Encoder is the core component used for Multi-View Video Coding (MVC). A common encoding structure is illustrated in Figure 6, featuring a total of five viewpoints, each with a Group of Pictures (GOP) length of 9. Temporally, the structure adopts a hierarchical B-frame bi-directional prediction scheme, while along the viewpoint direction, it uses an IBP prediction structure.

**Figure 7:**
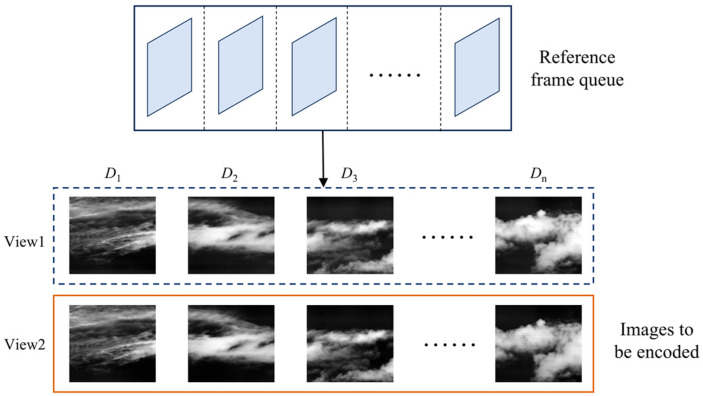
Large FOV infrared video to two-view MV video.

As shown in Figure 7, the high-resolution large FOV infrared image is divided into *n* sub-images, with distinct indices ${D_{k}=\{D}_{1},D_{2},D_{3},\ldots ,D_{n}\}$ assigned to the images based on their azimuth positions. In this study, the MV-Encoder is adapted for a dual-view configuration. The process begins by extracting the low-frequency components from the background reference queue of the previous push-scan period. Ordered by index, these components form the reference view, denoted as $V_{1}$. Simultaneously, the sequence of current low-frequency images to be encoded forms the coding view, denoted as $V_{2}$.

**Figure 8:**
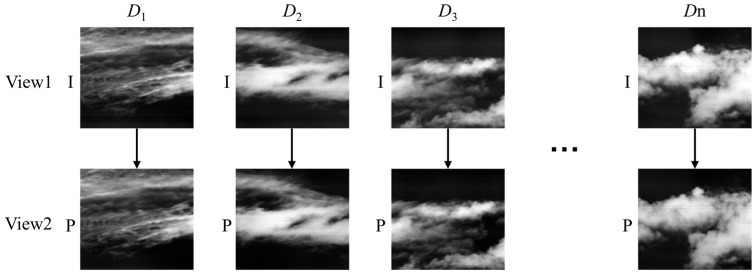
Multi-view background low-latency forward prediction structure.

Considering the weak correlation between temporally adjacent frames in ultra-high-resolution wide-field video, the encoding process is optimized to reduce encoding latency by adopting the following strategies. The prediction structure is shown in Figure 8:

(1) View 1 ($V_{1}$) Configuration:

View 1 is configured as I-frames, with each frame $L_{ref}^{\left(k\right)}\in V_{1}$ encoded independently within this viewpoint.

(2) View 2 ($V_{2}$) Configuration:

This view contains the encoded frames $L_{cur}^{\left(k\right)}\in V_{2}$ and is configured as low-latency P-frames, employing a forward prediction structure and referencing only a single image in the same azimuth from View 1:

$${\hat{L}}_{curr}^{\left(k\right)}=Predict(L_{curr}^{\left(k\right)}\;|\;L_{ref}^{\left(k\right)})$$

As View 1 consists of background reference frames originally stored in View 2 during the previous period, only the encoded bitstream from View 2 is written to the output file. This design maintains low latency and reduces computational costs by avoiding non-background images as reference frames, which often lead to ineffective inter-frame motion estimation.

### 3.4. High-Frequency Pixel-Wise Clustering Edge Prediction

This section presents a novel prediction framework for high-frequency subbands that integrates texture-adaptive clustering with edge pixel prediction. The proposed method significantly reduces inter-frame redundancy while maintaining low computational complexity compared to conventional motion estimation approaches.

#### 3.4.1. Problem Formulation and Notation

Let $f_{i}$ denote the current high-frequency subband to be encoded, with dimensions M×N pixels. The temporally corresponding background reference image is denoted as $f_{i-1}$. To enable localized processing, the image is partitioned into non-overlapping blocks of size 4 × 4 pixels.

For a current processing block $B_{i}$ with its top-left corner located at coordinates (x,y), we define the corresponding reference block $B_{i-1}$ from the background reference image. To capture sufficient contextual information for clustering and prediction, $B_{i-1}$ spans the region (x−3:x+6,y−3:y+6), encompassing 100 reference pixels. This extended window provides adequate spatial context for robust texture analysis and clustering operations. The relevant notation is defined in Table 2.

#### 3.4.2. Texture-Adaptive Reference Pixels Clustering

A Sorted Equipartition-based Single-Iteration Clustering (SESIC) method is employed to partition reference pixels into groups based on intensity similarity. A key innovation of our approach is the adaptive determination of the cluster count K based on local texture complexity. This ensures that regions with higher texture complexity receive finer quantization through more clusters, while homogeneous regions are efficiently represented with fewer clusters.

1. Texture Complexity Quantification

**Figure 9:**
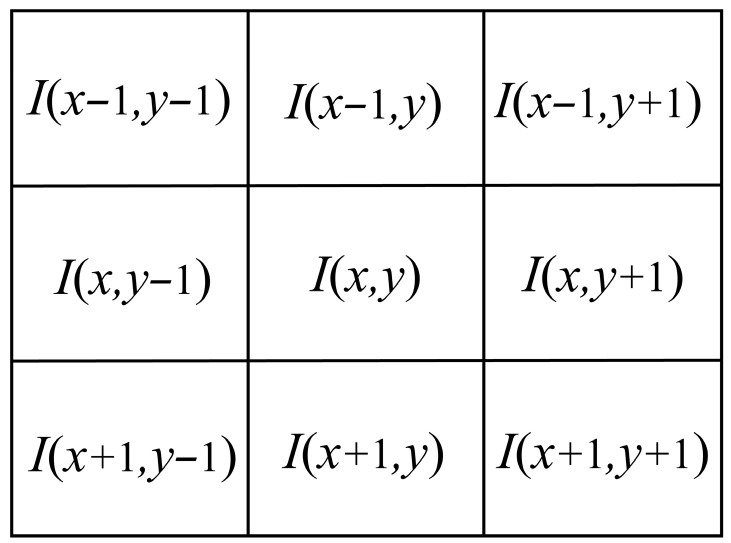
3 × 3 texture element.

Texture complexity is quantified using a fast differential method based on a 3 × 3 texture element [61]. The 3 × 3 texture elements are illustrated in Figure 9. For each pixel I(x,y) in the reference image, gradient magnitudes are computed along four orientations (Equations (4)–(7)):

Horizontal gradient:

(4) $$d_{h}=\left|I(x,y+1)-I(x,y-1)\right|$$

Vertical gradient:

(5) $$d_{v}=\left|I(x+1,y)-I(x-1,y)\right|$$

Left diagonal gradient (45°):

(6) $$d_{l}=\left|I(x+1,y+1)-I(x-1,y-1)\right|$$

Right diagonal gradient (135°):

(7) $$d_{r}=\left|I(x-1,y+1)-I(x+1,y-1)\right|$$

The pixel texture complexity metric D(x,y) is defined as the maximum gradient magnitude across all orientations (Equation (8)):

(8) $$D(x,y)=\max\{d_{h},d_{v},d_{l},d_{r}\}$$

The global average texture complexity for the entire reference image is computed as (Equation (9)):

(9) $$\mu_{t}^{\left(I\right)}=\frac{1}{M\times N}\sum_{x=1}^{M}\sum_{y=1}^{N}D(x,y)$$

Similarly, the local average texture complexity for each reference block is (Equation (10)):

(10) $$\mu_{t}^{\left(B\right)}=\frac{1}{100}\sum_{x=m-3}^{m+6}\sum_{y=n-3}^{n+6}D(x,y)$$

where (m,n) denotes the top-left coordinate of the current processing block.

2. Adaptive Cluster Number Determination

The number of clusters K is adaptively determined based on the relationship between local and global texture complexity (Equation (11)):

(11) $$K=10+I(\mu_{t}^{\left(B\right)}>\mu_{t}^{\left(I\right)})\cdot 5+I(\mu_{t}^{\left(B\right)}>2\mu_{t}^{\left(I\right)})\cdot 5$$

where I(⋅) is the indicator function. This formulation yields

(1) K=10 for regions with below-average complexity
(2) K=15 for regions with moderate complexity $\left(\mu_{t}^{\left(B\right)}>\mu_{t}^{\left(I\right)}\right)$
(3) K=20 for regions with high complexity ${(\mu }_{t}^{\left(B\right)}>2\mu_{t}^{\left(I\right)})$

3. Sorted Equipartition-based Single-Iteration Clustering

We propose a sorted equipartition-based single-iteration clustering (SESIC) algorithm as an efficient alternative to traditional K-means clustering. By exploiting the one-dimensional characteristic of pixel intensity values, it achieves near-optimal partitioning while substantially reducing computational complexity.

Let $X=\{x_{1},x_{2},\ldots ,x_{n}\}$ denote the set of n=100 pixel values in the reference block. we aim to partition them into K clusters. The algorithm proceeds in two main stages:

Stage 1: Sorted-Based Equipartition (SBE) Initialization

(1) Sorting: Sort the reference pixels in ascending order: $x_{\left(1\right)}\leq x_{\left(2\right)}\leq \cdots \leq x_{\left(N\right)}$, where $x_{\left(i\right)}$ denotes the i-th order statistic.
(2) Equipartitioning: The sorted sequence is divided the sorted sequence into K equal-sized partitions $C_{k}^{\left(0\right)}$ (Equation (12)):
  (12) $$C_{k}^{\left(0\right)}=\{x_{\left(i\right)}:(k-1)\cdot \lfloor N/K\rfloor <i\leq k\cdot \lfloor N/K\rfloor \},\;k=1,2,\ldots ,K$$
(3) Initial Center Calculation: The initial cluster centers $\mu_{k}^{\left(0\right)}$ are calculated as the mean of each partition: (Equation (13)):

(13) $$\mu_{k}^{\left(0\right)}=\frac{1}{\left|C_{k}^{\left(0\right)}\right|}\sum_{x\in C_{k}^{\left(0\right)}}x$$

Stage 2: Single-Iteration Optimization

To refine the initial estimates and approximate the optimal intra-cluster variance, a single round of cluster assignment and center update is performed.

(1) Reassignment: The dataset is partitioned into updated clusters $\left\{C_{1},\ldots ,C_{k}\right\}$ by assigning each data point $x_{i}$ to the nearest initial center (Equation (14)):
  (14) $$C_{k}=\left\{x_{i}\in X|k=\underset{j\in \{1,\ldots ,K\}}{argmin}\left|x_{i}-\mu_{j}^{\left(0\right)}\right|\right\}$$
(2) Update: Calculate the cluster center as the mean of each partition (Equation (15)):

(15) $$\mu_{k}=\frac{1}{\left|C_{k}\right|}\sum_{x\in C_{k}}x$$

4. Pixel-wise Clustering Edge Prediction

**Figure 10:**
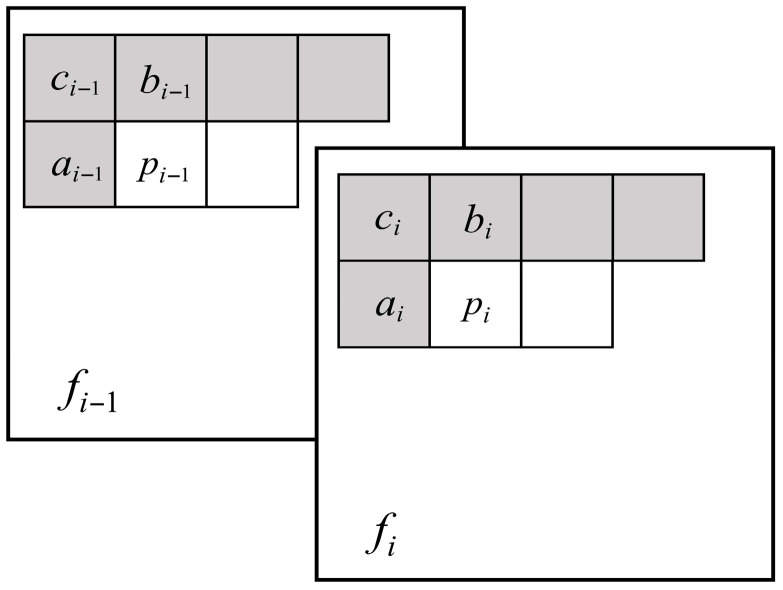
The context of pixel-wise prediction.

The pixel-wise prediction scheme effectively leverages both spatial (intra-frame) and temporal (inter-frame) correlations in infrared high-frequency components. The relevant context for this process is illustrated in Figure 10. We define the following notation:

Target pixel: $P_{i}$ (the pixel to be encoded)

Intra-frame reference pixels: $\left\{c_{i},a_{i},b_{i}\right\}$ (spatially neighboring pixels of $P_{i}$ in the current frame)

Inter-frame reference pixels: $\left\{p_{i-1},c_{i-1},a_{i-1},b_{i-1}\right\}$ (co-located pixels in the reference frame)

(1) Cluster-Based Prediction Values

For each reference pixel, we compute its cluster-based prediction by finding the nearest cluster center, the cluster center of ${\;\mu }_{k}$ is regarded as the clustering prediction value. For a pixel $c_{i-1}$(Equation (16)):

(16) $${\hat{c}}_{i-1}=\mu_{k},\;where\;k\;=\;{argmin}_{k=1,\ldots ,K}\|c_{i-1}-\mu_{k}{\|}^{2}$$

(2) Pixel Classification and Prediction

Pixels are classified as either smooth or non-smooth based on the local variance of intra-frame reference pixels (Equation (17)):

(17) $$\sigma_{i}^{2}=Var(\{c_{i},a_{i},b_{i}\})$$

Case1: Smooth Pixels $\left(\sigma_{i}^{2}<T_{hom},\mathrm{where}{\;T}_{hom}=35\right)$

For smooth regions, spatial correlation dominates, and prediction employs weighted averaging (Equation (18)):

(18) $${\hat{p}}_{i}=round\left(0.5\times p_{i-1}+0.2\times {\hat{p}}_{i-1}+0.3\times (a_{i}+b_{i}+c_{i})/3\right)$$

This formulation balances temporal prediction $p_{i-1}$, previously reconstructed values ${\hat{p}}_{i-1}$, and spatial context from neighboring pixels.

Case 2: Non-Smooth Pixels ($\sigma_{i}^{2}\geq T_{hom}$)

Non-smooth pixels undergo edge-adaptive prediction as described in the following subsection.

(3) Edge-Adaptive Prediction for Non-smooth pixels

For non-smooth pixels, the prediction strategy is adaptively adjusted based on the average absolute inter-frame difference (Equation (19)):

(19) $$\Delta_{avg}=mean\{|a_{i}-a_{i-1}|,|b_{i}-b_{i-1}|,|c_{i}-c_{i-1}|\}$$

Case 1: High or Low Temporal Correlation

If $\Delta_{avg}>T_{high}$ (significant temporal change) or $\Delta_{avg}<T_{low}$ (minimal change), where $T_{high}=0.95$ and $T_{low}=0.8$, the prediction adopts temporal copying:

$${\hat{p}}_{i}=p_{i-1}$$

Case 2: Moderate Temporal Correlation with Edge Analysis

When ${T_{low}\leq \Delta }_{avg}\leq T_{high}$, edge direction analysis is performed. The minimum difference between $p_{i-1}$ and its neighbors identifies the dominant edge orientation (Equation (20)):

(20) $$\Delta_{min}=\min\{|p_{i-1}-c_{i-1}|,|p_{i-1}-a_{i-1}|,|p_{i-1}-b_{i-1}|\}$$

**Figure 11:**
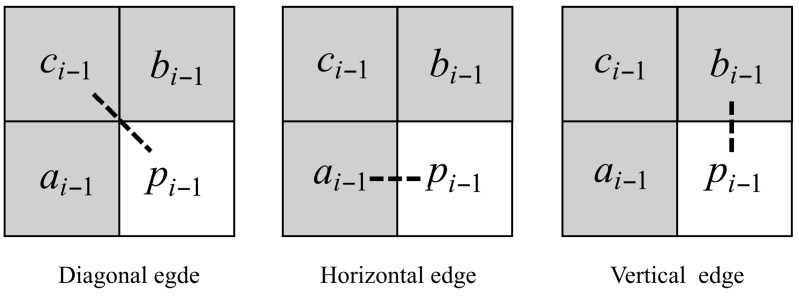
The non-smooth pixels edge orientation.

As illustrated in Figure 11, the neighbor yielding $\Delta_{min}$ indicates the edge direction:

$c_{i-1}$: diagonal edge

$a_{i-1}$: horizontal edge

$b_{i-1}$: vertical edge

Let $c_{i-1}$ denote the identified edge pixel. The prediction weights are computed as (Equation (21)):

(21) $$\lambda_{1}=1-0.02\times \Delta_{avg},\lambda_{2}=1-\lambda_{1}$$

The final prediction combines temporal and edge pixels (Equation (22)):

(22) $${\hat{p}}_{i}=round(\alpha *(\lambda_{1}\times p_{i-1}+\lambda_{2}\times ({\hat{p}}_{i-1}+{\hat{c}}_{i-1}+c_{i-1}))+(1-\alpha )*p_{i-1})$$

where α=1 if $\Delta_{min}\leq t,t=6$, else α=0.

The detailed implementation of the prediction method proposed in this section is presented in Algorithm 2.

   **Algorithm 2**: Pixel-wise clustering edge prediction for high-frequency components   **Input**: Target pixel $p_{i}$ to be encoded              Intra-frame reference pixels: $\left\{c_{i},a_{i},b_{i}\right\}$               Inter-frame reference pixels: $\left\{p_{i-1},c_{i-1},a_{i-1},b_{i-1}\right\}$              Cluster centers: $\mu =\{\mu_{1},\mu_{2},\ldots ,\mu_{K}\}$               Thresholds: $T_{hom}=35$ (smooth/non-smooth classification), $T_{low}=0.8$;               $T_{high}=0.95$ (temporal correlation), t=6 (edge difference threshold)   **Output**: Predicted value ${\hat{p}}_{i}$          35.    // Step 1: Compute cluster-based prediction values          36.    **for** each reference pixel ${r\in \{c}_{i-1},a_{i-1},b_{i-1}\}$
**do**          37.              $\hat{r}\leftarrow \mu_{k}$, where $k={argmin}_{k=1,\ldots ,K}\|r-\mu_{k}{\|}^{2}$          38.    **end for**          39.    // Step 2: Pixel classification based on local variance          40.    $\sigma^{2}\leftarrow Var(\{c_{i},a_{i},b_{i}\})$          41.    **if**
$\sigma^{2}<T_{hom}$
**then**          42.          // Case 1: Smooth pixel-weighted averaging prediction          43.          ${\hat{p}}_{i}\leftarrow round\left(0.5\times p_{i-1}+0.2\times {\hat{p}}_{i-1}+0.3\times (a_{i}+b_{i}+c_{i})/3\right)$          44.    **else**          45.          // Case 2: Non-smooth pixel edge-adaptive prediction          46.          $\Delta_{avg}\leftarrow mean\{|a_{i}-a_{i-1}|,|b_{i}-b_{i-1}|,|c_{i}-c_{i-1}|\}$          47.          **if**
$\Delta_{avg}>T_{high}$
**or**
$\Delta_{avg}<T_{low}$
**then**          48.                ${\hat{p}}_{i}\leftarrow p_{i-1}$                                                                  // High/Low temporal correlation: temporal copy          49.          **else**                                                          // Moderate correlation: edge direction analysis          50.                ${∆}_{c}\leftarrow |p_{i-1}-c_{i-1}|$          51.                ${∆}_{a}\leftarrow |p_{i-1}-a_{i-1}|$          52.                ${∆}_{b}\leftarrow |p_{i-1}-b_{i-1}|$          53.                $\Delta_{min}\leftarrow min({∆}_{c},{∆}_{a},{∆}_{b})$          54.                **if**
$\Delta_{min}={∆}_{c}$
**then**                              // Select edge pixel based on minimum difference          55.                      $e\leftarrow c_{i-1}$; $\hat{e}\leftarrow {\hat{c}}_{i-1}$                      // diagonal edge          56.                **else if**
$\Delta_{min}={∆}_{a}$
**then**          57.                      $e\leftarrow a_{i-1}$; $\hat{e}\leftarrow {\hat{a}}_{i-1}$                      // horizontal edge          58.                **else**          59.                      $e\leftarrow b_{i-1}$; $\hat{e}\leftarrow {\hat{b}}_{i-1}$                      // vertical edge          60.                **end if**          61.                $\lambda_{1}\leftarrow 1-0.02\times \Delta_{avg},\lambda_{2}\leftarrow 1-\lambda_{1}$   // Compute adaptive weights          62.                **if**
$\Delta_{min}\leq t$
**then**                            // Final edge-adaptive prediction          63.                     ${\hat{p}}_{i}\leftarrow round(\lambda_{1}\times p_{i-1}+\lambda_{2}\times ({\hat{p}}_{i-1}+\hat{e}+e)$          64.                **else**          65.                     ${\hat{p}}_{i}\leftarrow p_{i-1}$          66.                **end if**          67.          **end if**          68.    **end if**

#### 3.4.3. Adaptive Directional Residual Prediction

**Figure 12:**
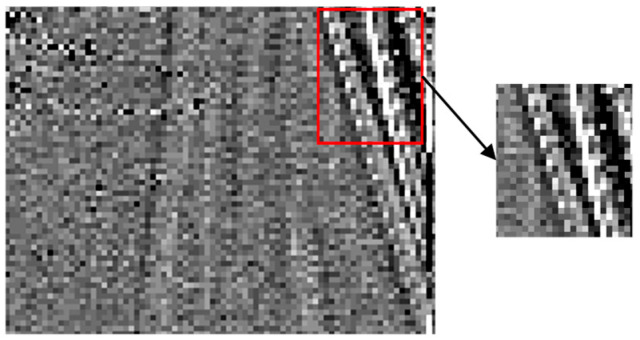
The predicted residual image.

Prediction residuals of video sequences typically exhibit structured edge features, especially in the vicinity of moving objects or textured regions (Figure 12). To further reduce residual energy, we introduce an adaptive directional prediction technique that leverages these structural characteristics.

1. Residual Block Analysis

**Figure 13:**
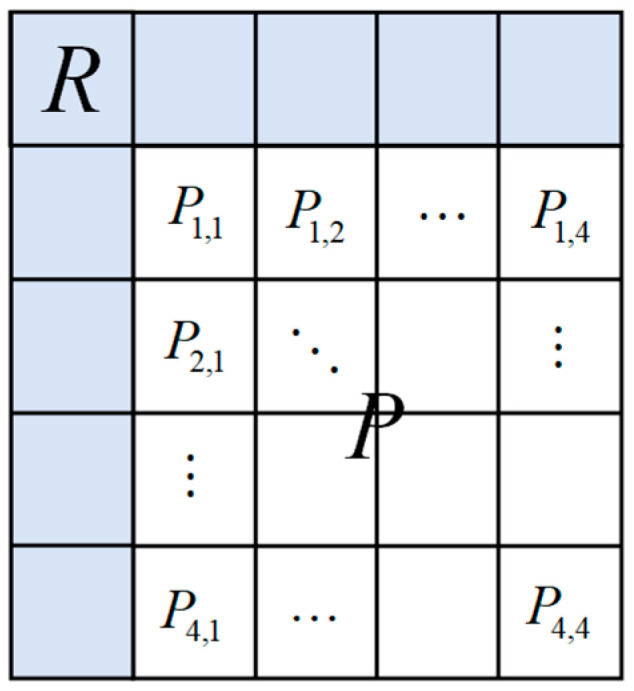
Reference pixels and pixels to be predicted. *P*: target residual block; *R*: set of 9 neighboring reference residual pixels.

Let P denote the 4 × 4 target residual block and R the set of 9 neighboring reference residual pixels (Figure 13). The decision to apply directional prediction is based on the mean absolute residual value (Equation (23)):

(23) $$R_{m}=\frac{1}{9}\sum abs(R(:,:))$$

Directional prediction is applied only when $R_{m}>T_{m}$ (where $T_{m}=8$); otherwise, the original residuals are retained. This adaptive threshold prevents unnecessary processing in low-energy regions where directional prediction offers minimal benefit.

2. Multi-Directional Residual Computation

**Figure 14:**
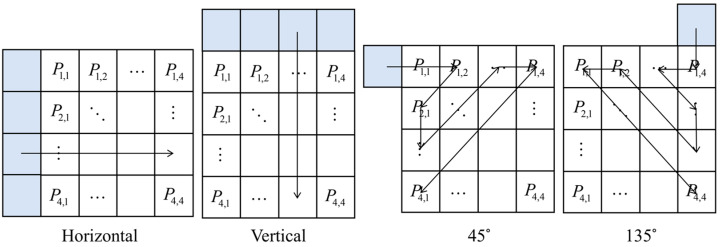
Prediction direction of the residual block. Arrows indicate the prediction direction. The blue regional pixels are derived from the *R* region.

Each directional prediction utilizes distinct sets of reference pixels ($RP_{h}$, $RP_{\nu }$, $RP_{45}$, and $RP_{135}$), which are derived from the P region and part of the R region (as shown in Figure 14). Residuals are computed for the four directions using Equations (24)–(27).

Horizontal prediction:

(24) $$G_{h}(:,j)=P(:,j)-RP_{h}(:,j),1\leq j\leq 4$$

Vertical prediction:

(25) $$G_{\nu }(j,:)=P(j,:)-RP_{\nu }(j,:),1\leq j\leq 4$$

45° diagonal prediction: (with zigzag scanning)

(26) $$G_{r}(j)=zigzag(P(j))-zigzag(RP_{45}(j)),1\leq j\leq 16$$

135° diagonal prediction: (with zigzag scanning)

(27) $$G_{d}(j)=zigzag(P(j))-zigzag(RP_{135}(j)),1\leq j\leq 16$$

The zigzag scan reorders 2D block elements into a 1D sequence following diagonal traversal, enabling efficient 1D differential prediction along diagonal orientations.

3. Optimal Direction Selection

The optimal prediction direction is selected based on energy minimization. The original residual energy is (Equation (28)):

(28) $$E_{ori}=\sum P^{2}$$

The post-prediction energy for each direction l∈{h,v,r,d} is (Equation (29)):

(29) $$E_{pred}^{\left(l\right)}=\sum G_{l}^{2}$$

The optimal direction m corresponds to minimum post-prediction energy (Equation (30)):

(30) $$m={argmin}_{l\in \{h,v,r,d\}}E_{pred}^{\left(l\right)}$$

The predicted residual block is adopted only if it reduces energy (Equation (31)):

(31) $$\hat{P}=\left\{\begin{array}{lll} G_{m}, & if\;E_{pred}^{\left(m\right)}<E_{ori} & \\ P, & otherwise & \end{array}\right.$$

4. Side Information Encoding

Directional prediction requires transmission of side information to enable decoder reconstruction:

A 1-bit flag indicates whether directional prediction is applied:

If flag = 1: Directional prediction is applied to the block, with a 2-bit code used to specify the selected direction (00: horizontal, 01: vertical, 10: 45°, 11: 135°).

If flag = 0: No directional prediction is performed, and the block retains its original values.

#### 3.4.4. Integration with JPEG-LS Framework

1. JPEG-LS baseline predictor

**Figure 15:**
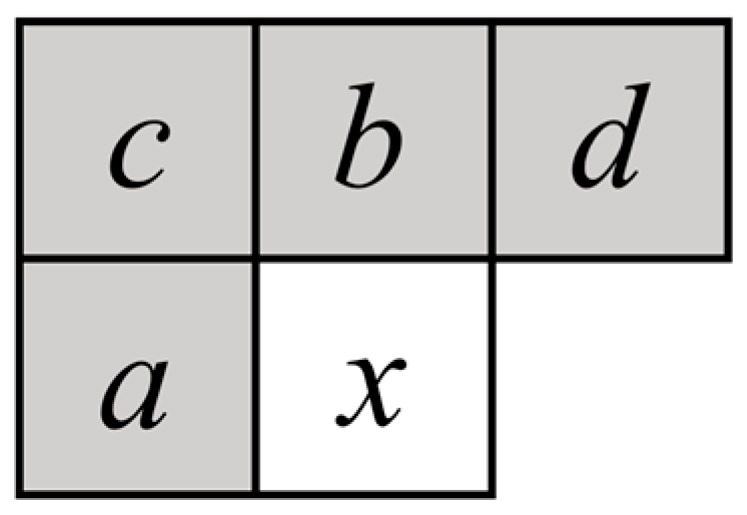
The context model of the pixel to be encoded.

The context model of the pixel to be encoded in JPEG-LS is shown in Figure 15. The current pixel value x and its four neighboring pixels are denoted as {a,b,c,d}. The corresponding reconstructed pixel values are represented as {Ra,Rb,Rc,Rd}. The prediction value under the regular mode is given by Equation (32).

(32) $$p_{x}=\left\{\begin{array}{lll} \min(Ra,Rb),Rc\geq \max(Ra,Rb) & & \\ \max(Ra,Rb),Rc\leq \min(Ra,Rb) & & \\ Ra+Rb-Rc,otherwise & & \end{array}\right.$$

2. Proposed Predictor Integration

**Figure 16:**
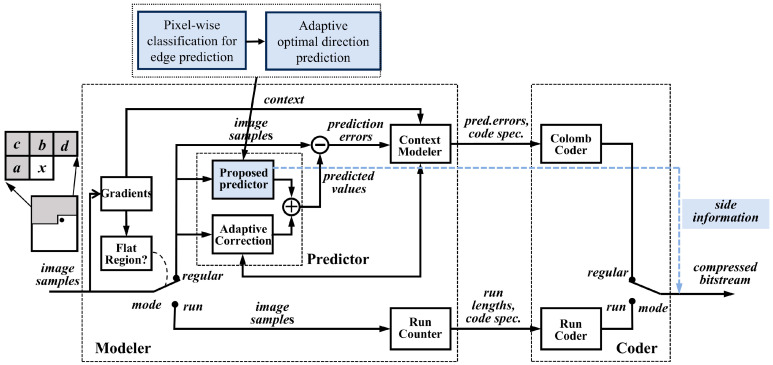
High-frequency subband predictor encoder.

The prediction mode of JPEG-LS is relatively simple and offers limited redundancy reduction performance in regions with complex textures. In this study, as shown in Figure 16, the proposed high-frequency predictor, which incorporates edge clustering and adaptive residual direction prediction, is integrated into JPEG-LS, replacing the fixed predictor to improve compression efficiency. Additionally, the side information necessary for optimal directional prediction is directly embedded in the bitstream file.

### 3.5. Computational Complexity Analysis

We introduce a hybrid prediction framework in which low- and high-frequency components are encoded using distinct strategies. The low-frequency component is downsampled to 1/8 resolution, substantially reducing the overhead of motion estimation. Conversely, high-frequency components utilize a pixel-wise classification edge prediction scheme. By avoiding redundant search operations, this scheme not only achieves significant complexity reduction compared to traditional motion estimation-based approaches but also enhances prediction accuracy by directly exploiting temporal correlations. A computational complexity analysis for a 4 × 4 high-frequency block is presented in Table 3.

## 4. Experimental Results

### 4.1. Datasets

**Figure 17:**
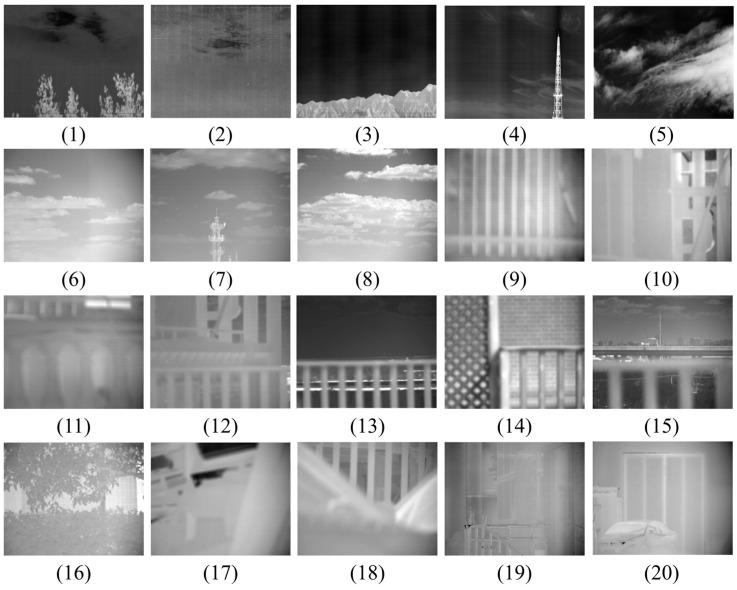
Partial original images of large FOV infrared videos with different resolutions: (**1**,**2**) 1024 × 896 pixels; (**3**,**4**) 2048 × 896 pixels; (**5**) 4096 × 896 pixels; (**6**–**20**) 640 × 512 pixels.

Large FOV infrared videos feature ultra-high resolutions and massive data volumes, with their panoramic images encompassing diverse and complex scenes. To thoroughly evaluate the compression performance of various methods across different scenarios, each test video is derived from segments of the large FOV infrared videos, forming a dataset of 20 test videos (Figure 17). Comparative experiments are then performed on these 20 infrared videos to compare the proposed method with other compression methods. Among them, Videos 1–5 consist of 8 push-scan cycles, with each cycle containing 10 sub-images; Videos 6–20 consists of 8 push-scan cycles, with each cycle containing 20 sub-images. The datasets and code generated during the current study are available from the corresponding author on reasonable request.

### 4.2. Performance Comparison and Configurations

Although H.266/VVC [16] offers superior compression efficiency, existing studies [22,23] indicate that the encoding overhead of H.266/VVC is significantly higher than that of H.264/AVC and H.265/HEVC. Such extreme latency is incompatible with the processing constraints of large-format infrared video systems. Therefore, H.264/AVC and H.265/HEVC are selected as the most representative baselines to benchmark the proposed algorithm’s efficiency.

To evaluate compression performance, the following compression methods and tools are compared: MV-HEVC [36], H.265/HEVC-Inter (inter-frame prediction) [14], HEVC-Intra (intra-frame prediction), H.264/AVC-Inter (inter-frame prediction) [13], H.264-Intra (intra-frame prediction), JPEG2000 [15], JPEG-XT Profile-C with Reinhard global tone-mapping [16], and PNG. All of the above methods employ lossless compression.

Encoder configurations: MV-HEVC: HTM16.3 [62]; H.265/HEVC: HM16.9 [63]; H.264/AVC: JM19.0 [64]; JPEG2000: OpenJPEG2.5.0 [65]; JPEG-XT: [66]. In all inter-frame predictions, one reference frame is selected.

All experiments were conducted on a platform equipped with an Intel Core i7-8750H CPU (2.1 GHz) and 8 GB of RAM, running Windows 10 (64-bit). The proposed algorithm was implemented in C/C++ without explicit parallelization. For a fair complexity comparison, all comparative methods were evaluated using their respective reference software.

### 4.3. Evaluation Metrics

The encoding performance of the different methods is evaluated using the structural similarity index (SSIM) of the reconstructed images compared to the original images (Equation (33)), the compression ratio (CR) (Equation (34)), the bits per pixel (BPP) (Equation (35)), and the encoding time metrics.

(33) $$SSIM=\frac{\left(2\mu_{I}\mu_{I^{\prime }}+C_{I})(2\sigma_{II^{\prime }}+C_{I^{\prime }}\right)}{\left(\mu_{I}^{2}+\mu_{I^{\prime }}^{2}+C_{I})(\sigma_{I}^{2}+\sigma_{I^{\prime }}^{2}+C_{I^{\prime }}\right)}$$

where $\mu_{I}$ and $\mu_{I^{\prime }}$ represent the mean values of the original image I and the reconstructed image $I^{\prime }$, respectively;${\;\sigma }_{I}$ and $\sigma_{I^{\prime }}$ denote the variances of the original image and the reconstructed image; and $\sigma_{II^{\prime }}$ indicates the covariance between the original image and the reconstructed image. Constants: $C_{I}=(K_{1}L{)}^{2},C_{I^{\prime }}=(K_{2}L{)}^{2}$.

(34) $$CR=\frac{Original\;image\;size}{Compressed\;image\;size}$$

(35) $$BPP=\frac{Compressed\;image\;size}{Image\;dimensions}$$

### 4.4. Results Analysis

(1) SSIM analysis

**Figure 18:**
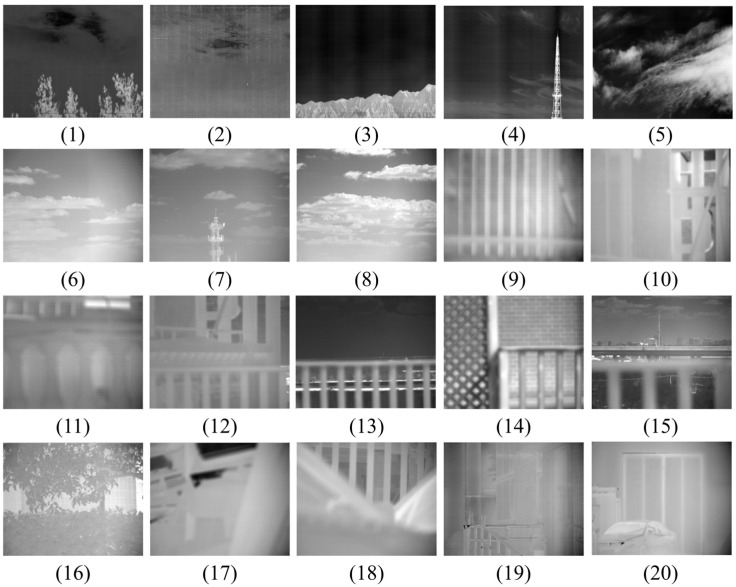
Partially reconstructed images of large FOV infrared videos with different resolutions: (**1**,**2**) 1024 × 896 pixels; (**3**,**4**) 2048 × 896 pixels; (**5**) 4096 × 896 pixels; (**6**–**20**) 640 × 512 pixels.

The reconstructed images after decoding are presented in Figure 18. The SSIM between decoded images and original images is used to evaluate whether the decoded images achieve perfect reconstruction. If SSIM = 1, it indicates that the original and decoded images are identical.

We calculated the SSIM between the reconstructed and original images for all test video sequences, and the results consistently show an SSIM of exactly 1.0 across all cases. This demonstrates that the proposed method achieves perfect image reconstruction, thus meeting the requirements of lossless compression.

(2) CR, BPP, and encoding time analysis

**Figure 19:**
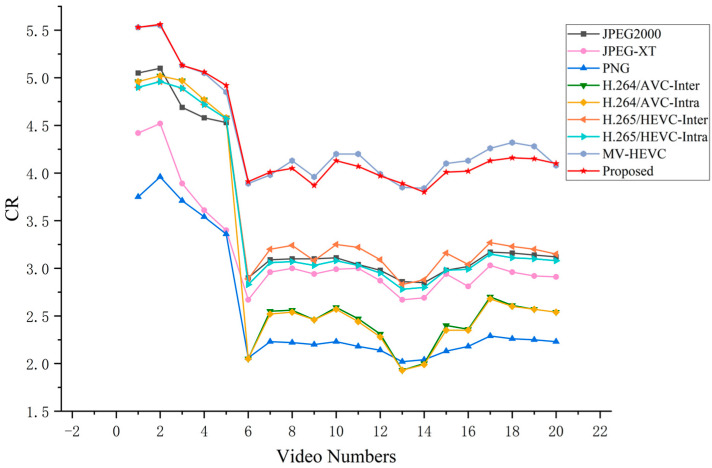
The CR of different methods across test videos.

**Figure 20:**
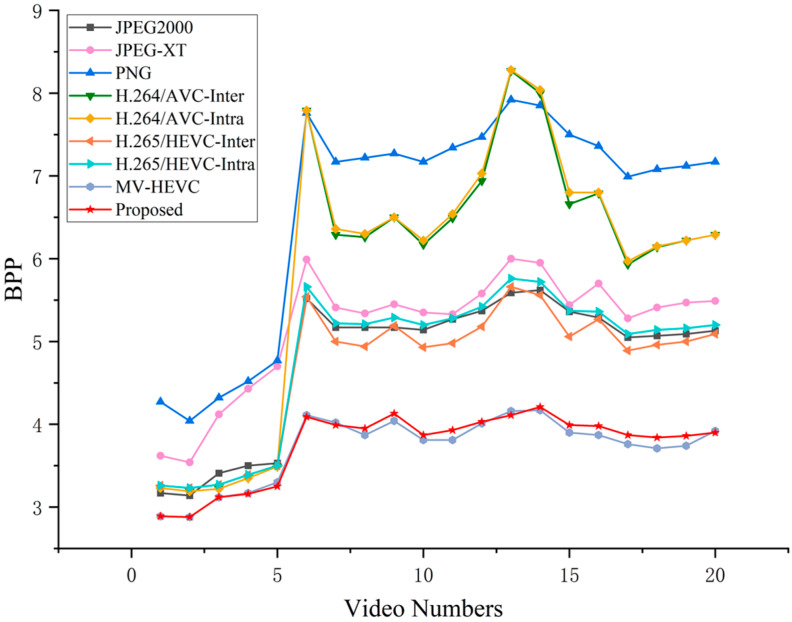
The BPP of different methods across test videos.

As shown in Figure 19 and Figure 20, the CR and BPP comparisons for each individual test video are presented, respectively. Table 4 summarizes the average CR, BPP, encoding time, and CR/Time of different compression methods across all test large FOV infrared video sequences. A higher CR and lower BPP indicate better compression efficiency. The proposed algorithm’s encoding time includes all essential steps of the proposed compression framework, such as wavelet transformation, background reference queue construction, prediction, and entropy coding.

- Compression Efficiency: The proposed method achieves a CR of 4.32 and a bpp of 3.75, which are comparable to the best-performing MV-HEVC (CR: 4.37, BPP: 3.71). Both methods significantly outperform conventional video codecs, with CR improvements of 22.0% and 23.4% over HEVC-Inter, and 43.0% and 44.7% over H.264-Inter, respectively. Image compression methods (JPEG2000, JPEG-XT, PNG) show inferior performance due to their inability to exploit temporal redundancy. The superiority of both the proposed method and MV-HEVC over conventional codecs stems from their effective exploitation of temporal redundancy. However, the proposed method distinctly outperforms MV-HEVC in efficiency by applying tailored predictive encoding methods to distinct frequency components.
- Inter- and Intra- Mode Performance Analysis: A noteworthy observation is that the inter-frame and intra-frame coding modes yield nearly identical compression performance for both H.264 (CR: 3.02 vs. 3.01) and HEVC (CR: 3.54 vs. 3.45). This phenomenon suggests that conventional block-based motion estimation techniques are inadequate for exploiting temporal redundancy in large FOV infrared video sequences. Due to limitations in the number of reference frames, long-term background correlations cannot be effectively captured, which degrades the performance of Inter-mode coding to a level comparable to Intra-mode.
- Computational Efficiency: The proposed method demonstrates a significant advantage in encoding speed, requiring only 0.8 s per frame. Compared to MV-HEVC, which achieves similar compression performance, the proposed method reduces encoding time by 94.9%. The speedup reaches 96.1% compared to HEVC-Inter and 71.3% compared to H.264-Inter. In terms of computational complexity, motion estimation typically accounts for over 70% of the total encoding time in traditional codecs like HEVC. This efficiency gain is attributed to the proposed hybrid prediction method, which obviates the need for extensive inter-frame motion estimation, thereby significantly reducing encoding time.
- Overall Evaluation: The CR/Time efficiency metric (compression ratio divided by encoding time) demonstrates that the proposed method achieves the best balance between compression performance and computational cost, with an efficiency score of 5.40, which is 19.3 times higher than MV-HEVC (0.28) and 31.8 times higher than HEVC-Inter (0.17). This makes the proposed method particularly suitable for real-time infrared video processing in embedded systems with limited computational resources.

## 5. Conclusions

This study proposes a transform-domain hybrid prediction method tailored for lossless compression of large FOV infrared videos, addressing the trade-off between compression efficiency and computational complexity in traditional codecs. Specifically, two-view background frame reference encoding is introduced for the low-frequency subband to effectively eliminate background redundancies. For the high-frequency subband, SESIC-based pixel-wise clustering prediction is performed without side information, avoiding traditional inter-frame motion estimation and significantly reducing encoding time. Additionally, optimal directional prediction is applied to the high-frequency prediction residuals to further reduce residual energy.

Comparative experiments confirm that the proposed method achieves compression performance comparable to MV-HEVC (CR: 4.32 vs. 4.37) while reducing encoding time by 94.9%, with a CR/Time metric 19.3 times higher than MV-HEVC, making it suitable for real-time applications in resource-constrained embedded systems. This work provides a new solution for alleviating data transmission and storage burdens in large FOV infrared imaging technology and enhances the practical application potential and efficiency of wide-field imaging technology in various fields.

Currently, the algorithm is validated in a software environment, necessitating further optimization for deployment on specific resource-constrained hardware platforms. Future work will focus on porting the framework to embedded systems to ensure real-time field performance, while simultaneously exploring lightweight deep learning techniques to further enhance prediction accuracy and environmental adaptability.

## Figures and Tables

**Table 1:** Impact of decomposition levels on prediction MAD.

Level	0	1	2	3	4
MAD	4.01	3.15	2.94	2.88	2.87
∆		−0.86	−0.21	−0.06	−0.01

**Table 2:** Texture-adaptive clustering notation definitions.

Symbol	Description
$f_{i}$	Current high-frequency subband data
$f_{i-1}$	Background reference image
$B_{i}$	Current 4 × 4 processing block
$B_{i-1}$	Extended reference block (10 × 10)
K	Number of clusters
$\mu_{k}$	Center of cluster *k*

**Table 3:** The computational complexity analysis for a 4 × 4 high-frequency block.

Operation	Description
Texture complexity computation	O(N)
Sorted-Based Equipartition clustering	O(N∗logN+N∗K)
Pixel-wise prediction	O(B∗logK)
Directional residual prediction	O(1)
** Total **	O(N∗logN)

Note: *N* = 100 is the reference block size, *B* = 16 is the number of pixels in a 4 × 4 block, and *K* denotes the number of clusters.

**Table 4:** Compression performance and encoding time comparison of different methods.

Methods	JPEG2000	JPEG-XT	PNG	H.264 -Inter	H.264 -Intra	HEVC -Inter	HEVC -Intra	MV -HEVC	Proposed
CR	3.48	3.16	2.55	3.02	3.01	3.54	3.45	4.37	4.32
BPP	4.79	5.18	8.16	5.86	5.89	4.7	4.84	3.71	3.75
Time/s				2.79	1.44	20.49	2.93	15.71	0.8
CR/Time				1.08	2.09	0.17	1.18	0.28	5.4

## Data Availability

The datasets used in this study is available on request from the authors.
